# A Case of Abscess of the Antrum Highmorianum, and Caries of the Outer Wall

**Published:** 1848-10

**Authors:** J. Weatherby

**Affiliations:** Dentist


### ARTICLE VI.

### *A Case of Abscess of the Antrum Highmorianum, and Caries of the Outer Wall.* By. J. WEATHERBY, Dentist.

September 13th, 1848, I was called to see Mrs. V——, aged about forty years. On examining her mouth, I found that the first and second superior molares on the right side, had been extracted some time before; the dens sapientiæ, the second bicuspis and cuspidatus decayed off to the gums; the first bicuspis sound, but very loose; the root of the cuspidatus very loose, and thick white matter oozing down the side of it. She informed me that it had been running twelve days; that before it broke, the pain was agonizing for several days, her face swelled so as to close her eye; when it broke it was very offensive; it broke in the nose and on the side of the root of the cuspidatus.

I suspected that the disease was in the antrum. I extracted the root of the cuspidatus, and introducing a probe I found the floor of the antrum destroyed, the anterior wall carious, and a piece of it loose and detached from the other bone. I then extracted the root of the second bicuspis, and perforated the floor of the antrum, making the orifice large enough to admit a small goose-quill; introducing a probe, I found the cavity empty, except above the first bicuspis and cuspidatus, where the lower part was covered with flesh, much inflamed and very sensitive. With a syringe, I injected warm water through the opening in the floor of the antrum, which passed out through the nose. I then extracted the first bicuspis. The apex of the root was very much diseased.

I made a weak solution of pulverized galls, nitrate of silver, tincture of myrrh and an elixir made of sulphuric ether, tannin and sulphate of morphia; with which I cleansed the parts twice a day, by means of a syringe.

In about ten days, the cavity was filled up and the orifice made through the floor of the antrum closed.

Sept. 29th.—The loose bone was removed by a slight effort; in about five days suppuration ceased and the patient was discharged.

Oct. 28th.—There were no symptoms of a return of the disease.

*Easton, Md., Nov. 20th,* 1848.

